# 1610. Adherence to Antiretroviral Therapy and Its Effect on Quality of Life among Persons with HIV in the United States using Real-World Data

**DOI:** 10.1093/ofid/ofad500.1445

**Published:** 2023-11-27

**Authors:** Fritha Hennessy, Girish Prajapati, Phoebe Salmon, Tim Holbrook, Bekana K Tadese

**Affiliations:** Adelphi Real World, Bollington, United Kingdom, Bollington, England, United Kingdom; Merck & Co., Inc., Rahway, New Jersey; Adelphi Real World, Bollington, United Kingdom, Bollington, England, United Kingdom; Adelphi Real World, Bollington, United Kingdom, Bollington, England, United Kingdom; Merck & Co., Inc., Rahway, New Jersey, United States, North Wales, Pennsylvania

## Abstract

**Background:**

Adherence to antiretroviral therapy (ART) is critical for persons with HIV (PWH) for achieving durable virologic suppression and minimizing drug resistance. This study aimed to characterize ART adherence and its effect on quality of life (QoL) among PWH using real-world data.

**Methods:**

Data were drawn from the Adelphi HIV Disease Specific Programme™, a cross-sectional survey of physicians and PWH in the U.S., collected between July-2021 and March-2022. At consultation, each of 60 physicians reported demographics, clinical characteristics, ART adherence and satisfaction on 10 consenting PWH. These PWH were then invited to complete questionnaires reporting QoL (PozQol, EQ-5D). Physicians categorized adherence as completely adherent (C-Adh), mostly adherent (M-Adh), or less adherent (L-Adh). Comparisons were made using Chi squared test.

**Results:**

Of 578 PWH with physician-reported adherence status; mean [SD] age 44.9 [12.8] years, 70.4% cisgender male, 50.5% White. A total of 389 (67%) were C-Adh, 155 (27%) M-Adh, and 34 (6%) L-Adh. L-Adh (55.9%) and M-Adh (46.5%) PWH had the higher proportion of ART side effects compared to C-Adh (29.3%); p< 0.001. Also, L-Adh (75.8%) and M-Adh (50.8%) were more likely to report current HIV-associated symptoms than C-Adh PWH (29.3%); p< 0.001. C-Adh PWH were perceived as very satisfied with their ART (68.4%), compared to M-Adh 39.4% and L-Adh 23.5%; p< 0.001. Reasons for PWH skipping ART included forgetting, difficulties integrating into routine, and ART side effects (Figure 1). QOL survey was completed by 229 PWH. QoL was higher for C-Adh PWH, measured by PozQol score mean, (C-Adh;45.2, M-Adh ;40.7, L-Adh;42.0; p< 0.05) and EQ-5D score mean (C-Adh; 0.91, M-Adh;0.77, L-Adh;0.66; p< 0.001).

**Figure:**
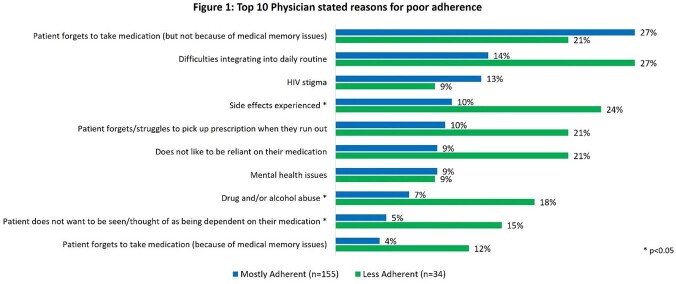

**Conclusion:**

About one-third of PWH who were not fully adherent to ART, and this was correlated with having poorer QoL. These findings highlight the need to better understand factors leading to sub-optimal ART adherence, to provide targeted interventions to improve quality of life.

**Disclosures:**

**Girish Prajapati, M.B.B.S., MPH** , Merck & Co., Inc.: Employee|Merck & Co., Inc.: Stocks/Bonds **Bekana K. Tadese, PhD, MPH**, Merck and Co Inc: Employee

